# Synthesis of titania/activated carbon composites for the synergistic adsorption and photocatalysis of lindane in aqueous solutions

**DOI:** 10.1007/s11356-025-36104-0

**Published:** 2025-02-25

**Authors:** Anastasia Stavrinou, Maria A. Theodoropoulou, Christos D. Tsakiroglou

**Affiliations:** 1 Foundation for Research and Technology Hellas, Institute of Chemical Engineering Sciences, Transport Phenomena and Porous Media Laboratory, Stadiou Str, Platani, 26504 Patras, Greece; 2 Department of Physics, University of Patras, 26504 Patras, Greece

**Keywords:** Adsorption, Activated carbon, Water treatment, Multi-compartment model, Lindane, Photocatalysis, Porous materials

## Abstract

Lindane (LIND) is an organochlorine pesticide, belonging to the persistent organic pollutants (POPs), which are bioaccumulative with detrimental effects on human health and environment. In the present work, activated carbon (AC) produced from coffee waste after activation with sodium hydroxide (NaOH) and pyrolysis at 800 °C (CWAC-NaOH-800) is tested as adsorbent of LIND, and as substrate for the synthesis of hybrid adsorbent/photocatalytic materials of titanium oxide with activated carbon (TiO_2_/AC) via the sol–gel method, aiming at the synergistic adsorption and photocatalysis of LIND. Three different TiO_2_/AC materials were synthesized at mass ratio of TiO_2_ to AC equal to 2.0 (material TiO_2_/AC-0.2), 4.0 (material TiO_2_/AC-0.1), and 8.0 (material TiO_2_/AC-0.05). As the TiO_2_ to AC mass ratio increases, the specific surface area and total pore volume of the composite materials decrease, the meso-/macro-porosity are reduced and a pore-and-throat microporous network is created within the agglomerates of TiO_2_ nanoparticles. The adsorption of LIND onto the CWAC-NaOH-800 is pH-independent and follows the Langmuir model with a maximum adsorption capacity equal to 9.74 mg/g. The adsorption mechanism is likely to be the hydrophobic interactions as generally ACs tend to repel water molecules and preferentially adsorb non-polar or hydrophobic compounds such as LIND. The mass transfer multi-compartment model was used to describe the LIND sorption dynamics in CWAC-NaOH-800 and TiO_2_/AC-0.1, by accounting for the film/pore/surface diffusion and instantaneous linear sorption, and it was found that the contribution fraction of surface diffusion and microporosity to the total LIND sorption was enhanced with the presence of TiO_2_, due to the decrease of meso-/macro-porosity. For the study of the adsorptive-photocatalytic capacity of composite materials, two experimental setups were tested and compared: an ultraviolet-A (UVA) oven with LEDs (power = 22 W, *λ* = 375 nm) radiating from the periphery to the center of the reactor, and a UVA lamp (power = 6 W, *λ* = 375 nm) radiating radially from the center to the reactor walls. The material TiO_2_/AC-0.1 showed LIND sorption capacity comparable to that of CWAC-NaOH-800 and photocatalytic performance better than that of TiO_2_ nanoparticles, due to the presence of AC and the “co-adsorption” effect. During the synergistic adsorption-photocatalysis process, the TOC removal efficiency reached 90% after 3 h of photocatalysis, which is indicative of the in situ regeneration of the material. From the inverse modeling of the adsorption-photocatalysis experiments, it was found that the kinetic constant of TiO_2_/AC-0.1 photocatalyst was highest in the experimental setup of the UVA lamp, showing a good balance between adsorptive and photocatalytic capacity.

**Graphical abstract:**

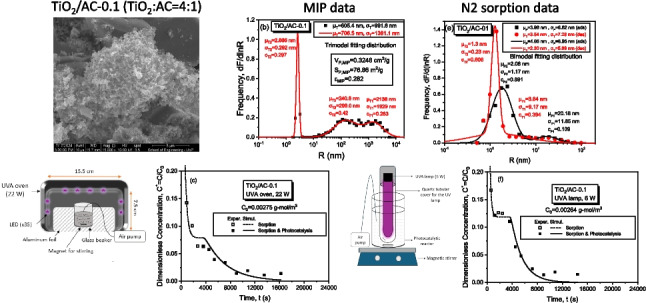

## Introduction

Organochlorine pesticides are one of the most harmful categories of pollutants, as many of them belong to the persistent organic pollutants (POPs) being bio-accumulative, having harmful effects on human health and on the environment and potential for long range transport (Sheriff et al. 2022). Lindane (LIND) or γ-hexachlorocyclohexane (C_6_H_6_Cl_6_) is an organochlorine insecticide, firstly introduced as a scabicide for human use in the 1950s (Elkady et al. 2013), having excessive application to vegetable and fruit production, as well (Li 1999). It is included in the list of the high priority pollutants of the environmental protection agency (EPA) (Sotelo et al. 2002) and is considered as a POP, being added to Annex A of the Stockholm Convention POP list (Sheriff et al. 2022). The guideline value of the acceptable limit of LIND in water, established by World Health Organization (WHO) is 0.002 mg/L (WHO 2011). POPs are connected with many health issues such as carcinogenicity, teratogenicity, and neurological problems (Khan et al. 2017). In addition, the presence of six chlorine atoms in the LIND molecule makes it a highly toxic compound characterized as an endocrine disruptor in the environment (Pant et al. 2014). For the aforementioned reasons, there is a great need for eco-friendly, efficient, and cost-effective technologies for the removal of these types of pollutants from contaminated water and soil.

Among the methods adopted for the removal of LIND, adsorption is very promising owing to its simplicity and effectiveness, as it usually provides stable mechanisms of complexation between adsorbate molecules and adsorbent. One of the most advantageous and versatile adsorbents that are widely reported in the literature, is activated carbon (AC) produced from agricultural wastes as it combines the high efficiency and the low cost of production (Stavrinou et al. 2023a, b). The large specific surface area, the high porosity, and in many cases, the variety of functional groups lead to the high adsorption capacity of ACs for a broad variety of pollutants. Sotelo et al. (2002) used the commercial activated carbon F-400 correlating its porous properties to the adsorption capacity, and Elkady et al. (2013) prepared activated carbon from date stones obtaining a maximum removal percentage 98.6%. Other types of adsorbents have also been used for the removal of LIND from aqueous solutions. Nguyen et al. (2020) synthesized aluminum hydroxide Al(OH)_3_ nanomaterials with surface modification by the anionic surfactant sodium dodecyl sulfate (SDS), achieving removal efficiency up to 93.68%. Mehmeti et al. (2022) used graphene and graphene oxide (synthesized by reducing graphene oxide with ascorbic acid) with corresponding efficiencies of 92.5% and 56.1%.

Nevertheless, adsorption is unable to decompose the pollutants, and therefore, the adsorbed pollutants have to be degraded with additional techniques. The advanced oxidation processes (AOPs) are known for their capability to decompose many categories of pollutants to less toxic or non-toxic substances, with the generation of reactive radical species (Khan et al. 2016). More specifically, metal oxide–based heterogenous photocatalysis is considered as one the most efficient AOPs. The fundamental principle of this technique is the generation of holes (h^+^), electrons (e^−^), and reactive oxygen species (ROS) such as superoxide radical (**·**O_2_^−^), hydroxyl radical (**·**OH), hydrogen peroxide (H_2_O_2_), and singlet oxygen (^1^O_2_) through light irradiation in the visible or ultraviolet region of the solar spectrum (Wang et al. 2022). The most popular semiconductor-photocatalyst is titanium dioxide (TiO_2_), which beyond of its effectiveness towards the removal of different types of pollutants, is stable, widely available, cheaper than other catalysts, and non-toxic (Lyu et al. 2014; MiarAlipour et al. 2018). The reaction mechanism is the following: the e^−^ in the valence band of TiO_2_ are transferred to the conduction band by generating h^+^ at the valence band, when a UV photon is adsorbed. The h^+^ react with water (H_2_O) or hydroxyl ions to form **·**OH and the e^−^ react with oxygen (O_2_) to form superoxide **·**O_2_^−^. The photo-induced ROS take part in oxidation–reduction reactions with the molecules of the pollutants and can achieve mineralization by producing CO_2_ and H_2_O (Qu et al. 2013; Dong et al. 2015). Senthilnathan and Philip synthesized successfully N-doped TiO_2_ photocatalysts which demonstrated satisfactory photocatalytic activity under visible light irradiation (Senthilnathan and Philip 2010). Khan et al. (2019) investigated the degradation of LIND by TiO_2_ photocatalyst activated by simulated solar light. They achieved removal efficiency 23% in 6 h, which increased to 64%, 89%, and 99% with the addition of hydrogen peroxide (H_2_O_2_), persulfate (S_2_O_8_^2−^), or both, respectively. Shah and Patel (2021) investigated the photocatalytic reduction of LIND using P25 (TiO_2_) in the presence of oxalic acid and Ethylenediaminetetraacetic acid (EDTA) as hole-scavengers and achieved complete reductive dechlorination in the presence of 1 mM EDTA in 90 min.

In spite of the abundant advantages of TiO_2_, there are some limitations that weaken its photocatalytic performance, including the agglomeration of nanoparticles, the limited light absorption due to the wide band gap, the rapid recombination of e^−^ and h^+^ (Fazal et al. 2020), and the poor affinity for hydrophobic organic compounds, which makes the adsorption of organic pollutants on its surface relatively slow (MiarAlipour et al. 2018). One way to overcome these drawbacks is the immobilization of TiO_2_ on a suitable support matrix. The incorporation of TiO_2_ nanoparticles in the structure of an adsorbent could reduce the limitations of both adsorption and photocatalysis, offering a synergistic effect on the degradation process. Several adsorbent supports for TiO_2_ have been reported in the literature, such as biochar (Fazal et al. 2020), clay (Mogyorósi et al. 2002; Paul et al. 2012), zeolite (Gomez et al. 2013; Liu et al. 2014), and AC (Xue et al. 2011; Martins et al. 2017; Amorós-Pérez et al. 2021; Ul Haq et al. 2023). Among these adsorbents, AC is considered a very efficient support because of its textural properties and robust structure. Martins et al. (2017) successfully synthesized TiO_2_/AC photocatalyst via the sol–gel method for the degradation of the antibiotic tetracycline from aqueous solutions. They concluded that the AC improved the photocatalytic properties of the material by increasing the specific surface area, providing useful structural features, lowering the band gap energy (*E*_*g*_) and increasing the kinetic constant compared to bare TiO_2_ and commercial photocatalyst P25 (Martins et al. 2017). Amorós-Pérez et al. (2021) have also applied the sol–gel method to synthesize TiO_2_/AC composites for the removal of the herbicide Diuron from water and found that the effectiveness of the material increases with the carbon content increasing, as a result of the combination of Diuron adsorption with photodegradation. Such a synergistic effect has been observed by Ul Haq et al. (2023) who investigated the degradation of hydrocarbon pollutants of oil refinery wastewater, and realized that the photocatalytic oxidation and adsorption by the TiO_2_/AC composite was advantageous compared to the individual adsorption and photocatalytic oxidation using bare AC and TiO_2_. The photocatalytic activity of TiO_2_/AC composite, calcinated at 700 °C, was tested, and compared with that of TiO_2_ Degussa P25 on the degradation of methylene blue (MB) in an aqueous solution under visible irradiation, and was found to be two times more active than TiO_2_-P25 (Slimen et al. 2011).

Despite the progress achieved on the use of TiO_2_/AC composites for the removal of various types of pollutants from water, their application to the degradation of emerging, recalcitrant, and persistent organic pollutants like LIND is still questionable. In the present work, sustainable and environmentally friendly materials are fabricated from biomass and tested as agents for water decontamination from persistent organic pollutants. Special emphasis is paid on the correlation of the properties of the AC and hybrid TiO_2_/AC with their performance as either adsorbent or adsorbent/photocatalyst, respectively, with regard to the degradation of LIND in aqueous solutions.

In the first part of the present work, the adsorption of LIND onto AC produced from coffee waste (CWAC) is studied by examining the effects of pH, initial LIND concentration, and contact time on the LIND removal efficiency, and correlating the properties of the pore structure of CWAC with the adsorption mechanisms. In addition, the multi-compartment model is used to describe the LIND sorption dynamics on the porous CWAC, by using the pore structure properties as input parameters. The model is used to simulate kinetic sorption tests and estimate the mass-transfer coefficients governing the rates of external and internal diffusive processes over the various compartments (external surface, meso-/macro-pore region, micro-pore region) of adsorbent with inverse modeling. At the second part of the work, the same AC is used to synthesize TiO_2_/AC composites via the sol–gel method, based on the hydrolysis of titanium (IV) isopropoxide (TTIP), and the synergistic adsorption and photocatalysis of LIND is studied. The TiO_2_/AC composites are synthesized at three mass ratios of AC to TiO_2_ by varying the AC dosage. For the photocatalytic experiments, two different setups are tested and evaluated: a UVA oven with LEDs surrounding the reactor, and a cylindrical UVA lamp inserted at the center of reactor, both emitting at 375 nm. Finally, the kinetic constant for LIND sorption and surface photocatalytic reaction are estimated with inverse modeling of experiments conducted at darkness and UV-radiation, and the most efficient composite material is selected.

## Materials and methods

### Chemicals

**Fig. 1:**
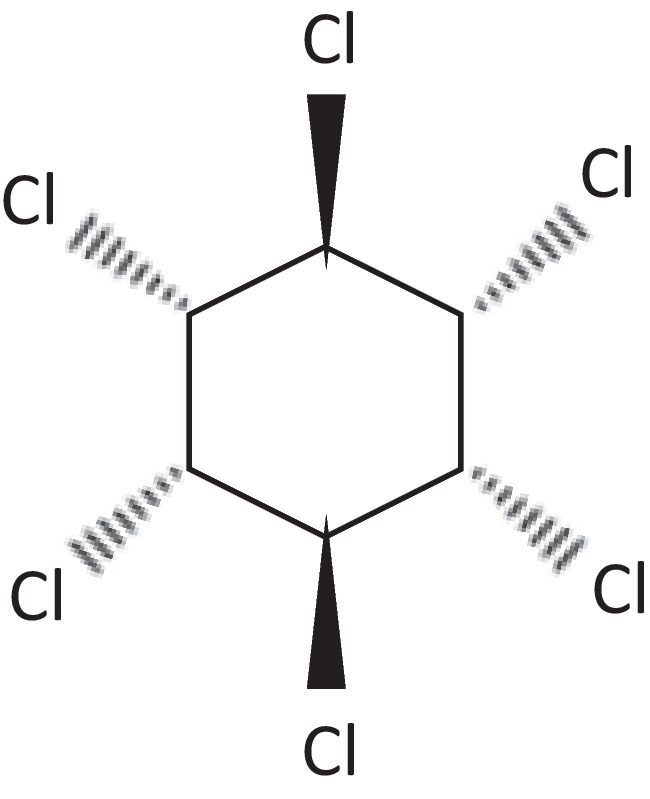
Chemical structure of LIND

LIND of purity 97% (Sigma-Aldrich) was the model pollutant (Fig. 1). Stock LIND solutions were prepared at concentration 100 mg/L using acetone (Honeywell, pesticide grade) as solvent and working solutions were prepared by diluting the stock solution in distilled water with sonication for 10 min at 40 °C. For the extraction of LIND, hexane was used (Honeywell, pesticide grade). Titanium (IV) isopropoxide (TTIP-Ti[OCH(CH_3_)_2_]_4_) (Sigma-Aldrich, ≥ 97%), absolute ethanol (Fluka, ≥ 99.8%), acetic acid (Honeywell, ≥ 99.8%), and nitric acid (Honeywell, ≥ 65%) were used for the preparation of TiO_2_/AC composites. Sodium hydroxide (Penta, NaOH) and hydrochloric acid (Honeywell, HCl) were used for pH adjustment. All chemicals used in the present work were of analytical grade.

### Preparation of TiO_2_/AC composites

**Fig. 2:**
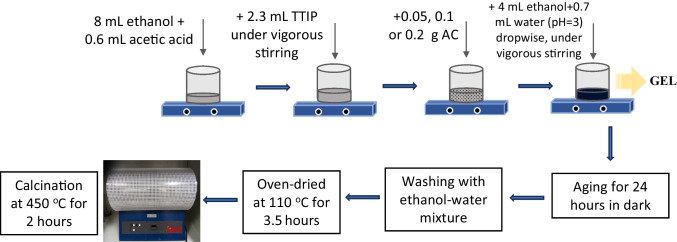
Flow-chart of the synthesis of TiO_2_/AC composites

The AC used for the synthesis of the composite was prepared from coffee waste pre-treated with NaOH (CWAC-NaOH-800), and the analytical procedure of its preparation is reported elsewhere (Stavrinou et al. 2023b). The TiO_2_/AC composites were synthesized based on the sol–gel method as it is shown in Fig. 2 and described below: TTIP (2.3 mL) was used as the precursor of TiO_2_ nanoparticles and was diluted in a mixture of 8-mL ethanol and 0.6-mL acetic acid which was stirred vigorously. The addition of TTIP to the ethanol-acetic acid mixture was carried out slowly by immersing the pipette inside the solution to avoid rapid hydrolysis of TTIP due to its exposure to the air humidity. After 5 min of stirring, 0.05, 0.1, or 0.2 g of AC were added (TiO_2_/AC-0.05, TiO_2_/AC-0.1, TiO_2_/AC-0.2). For the hydrolysis of the catalyst, a solution was prepared by mixing 4 mL of ethanol with 0.7 mL of water with pH equal to 3 (adjusted with HNO_3_ 0.1 N), and was added dropwise in the suspension of AC. After ~ 20 min, a homogenous gel was created. The gel was aged for 24 h, loosely covered in dark. Then, it was washed three times with 16 mL of ethanol–water solution (1:1) to remove the impurities and the TiO_2_ nanoparticles non-embedded in the TiO_2_/AC composite. The solid material was collected with centrifugation for 5 min at 6000 rpm. Afterwards, the material was dried in the oven at 110 °C for 3.5 h. Finally, it was calcined at 450 °C in an annular oven (Lenton), for 2 h at a rate of 10 °C/min under static air environment. The same procedure, without the addition of AC, was followed to synthesize pure TiO_2_ nanoparticles. The above processes yield TiO_2_ nanoparticles with a mass of 0.4 g.

### Physicochemical characterization of adsorbent/photocatalysts and analytical techniques

The textural properties of the TiO_2_/AC composites were analyzed with N_2_ adsorption/desorption isotherms which were measured at 77 K with a computer-controlled Quantachrome® ASiQwin™ instrument (AntonParr). Prior to N_2_ sorption tests, the samples were dried and evacuated. The specific surface area, ${S}_{BET}$, of the samples was calculated by applying the Brunauer–Emmett–Teller (BET) equation over low relative pressures of the N_2_ adsorption isotherm. The pore radius and throat radius distributions over the micropore/mesopore regions were determined by differentiating the N_2_ adsorption and desorption curve, respectively, with the aid of Barrett–Joyner–Halenda (BJH) method and ignoring any pore network topological effects (Stavrinou et al. 2023a). The pore structure of the materials over the macro-pore and meso-pore area was analyzed with mercury intrusion porosimetry (MIP) in a computer-controlled Quantachrome PoreMaster 60 instrument (Anton Parr). The pore and throat radius distributions, obtained from N_2_ sorption isotherms and Hg intrusion curve, were fitted with multi-modal (uni-, bi-, tri-) distributions, composed of log-normal component functions.

The crystallinity of the TiO_2_/AC composites was identified by X-ray diffraction with a Bruker D8 Advanced instrument operating with Ni filtered CuKα1 radiation in the range of  $2\theta$=10–80°. The average crystallite size was calculated by Scherrer’s equation (Eq. 1).

1 $$D=\frac{K\lambda }{\beta cos\theta }$$

where $D$ is the average crystallite size (nm); $K$ is a constant with a value of 0.9; $\lambda$ is the wavelength of the radiation source (λ = 0.154059 nm); $\beta$ is the full width at half maximum intensity (FWHM); and $\theta$ is the angle associated with the main peak corresponding to the studied phase ($2\theta$ values of 25.3° and 27.5° for anatase and rutile, respectively).

The morphology of the materials was examined by field-emission scanning electron microscopy (FE-SEM) with a FEI InspectTM F50 instrument using the SE-detector. The samples were deposited on carbon tape in order to provide a conduct path.

The amount of carbon in the composite materials was determined by thermogravimetric analysis (TGA) using a Waters Discovery TGA55 instrument. The analysis was carried out with the following conditions: air stream of 10 mL min^–1^, heating rate of 10 °C min^–1^, and temperature range of 10–800 °C.

### Adsorption of LIND onto the adsorbent CWAC-NaOH-800

The adsorption experiments were performed in glass vials on an overhead shaker operating at a speed of 1.5 rpm inside an incubator (Friocell) to ensure a constant temperature of 25 °C. Parametric analysis took place to investigate the effect of pH (2–10), the initial LIND concentration (0.1–8 mg/L), and the contact time (10–180 min) on the adsorption capacity on CWAC-NaOH-800. The sorption dynamics was investigated in depth by matching the transient responses of LIND concentrations of kinetic experiments, conducted at three different initial concentrations (0.76, 1.54, 3.10 mg/L), with the mass transfer multi-compartment model, and estimating the relevant mass-transfer coefficients. To determine the sorption isotherm, an adsorbent dosage of 0.01 g was mixed with 10 mL of LIND solutions. To examine the pH-dependence, and sorption kinetics, an adsorbent dosage of 0.002 g was mixed with 10 mL of LIND solutions to keep identical the AC mass in the adsorbent CWAC-NaOH-800 and composite TiO_2_/AC-0.1, and facilitate the comparison of the performance of the two materials.

At the end of the experiments, samples were collected, filtered with 0.45-μm syringe filters, extracted and analyzed with gas chromatography coupled with electron capture detector (GC-ECD, Shimadzu-2010). The liquid–liquid extraction method was followed. Extraction took place at 8-mL amber glass vials containing 1-mL LIND sample and 3-mL solvent (acetone-hexane 1:1). The mixture was sonicated for 20 min at 40 °C, stirred with vortex for 10 min and centrifuged for 5 min at 3000 rpm. The organic phase was collected at the top of the mixture, put into airtight amber glass vials, and injected into the GC-ECD with an AOC-20i autosampler. The recovery percentage of the extraction was about 85%. The conditions for LIND analysis were the following: the column was a mega 5-HT capillary column (5% phenyl and 95% methyl polysiloxane, 30 m × 0.25 mm, i.d., × 0.25-μm phase thickness); the injection port was held at 280 °C and operated in splitless mode; helium at constant flow of 1 mL/min was used as carrier gas; the column was held at 100 °C for 2 min, then heated to 190 °C at a rate of 18 °C/min and held for 5 min, and finally heated to 280 °C at a rate of 10 °C/min and held for 1 min; the ECD temperature was maintained at 290 °C (Karavasilis et al. 2022a). The amount of pollutant adsorbed at time $t$, ${q}_{t}$ (mg/g), and the adsorption capacity at equilibrium, ${q}_{e}$ (mg/g), were calculated by the following equations:

2 $${q}_{t}=\frac{{C}_{0}-{C}_{t}}{m}\times V$$

3 $${q}_{e}=\frac{{C}_{0}-{C}_{e}}{m}\times V$$

where ${C}_{0}$, ${C}_{t}$, and ${C}_{e }$(mg/L) are the LIND concentrations at time $0$, $t$, and equilibrium, respectively, $V$, is the volume of the LIND solution and $m$ is the mass of the adsorbent.

### Synergistic adsorption and photocatalysis of LIND with the TiO_2_/AC composites

**Fig. 3:**
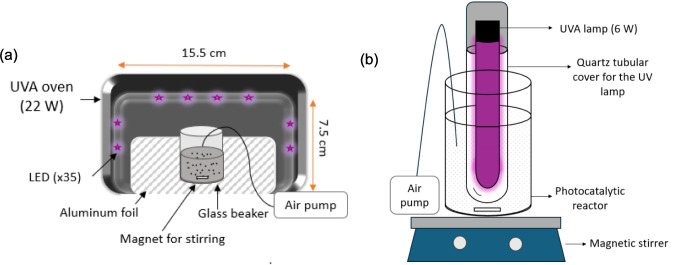
Schematic representation of the photocatalysis experimental setups: **a** UVA oven (22 W), **b** UVA lamp (6 W)

The experiments took place in the absence of light inside an incubator at constant temperature of 25 °C. Figure 3 shows the schematics of the two experimental setups which were used to investigate the effect of different conditions on the photocatalytic degradation of LIND. The UVA oven (Fig. 3a) has 35 LEDs placed across its lateral surface (dimensions 21.3 × 20 × 10 cm), and emitting at 375 nm at a total power of 22 W (Karavasilis et al. 2022b). The reactor, a glass beaker of 100 mL, is placed inside the UVA oven, and an air pump is used to supply air in the liquid phase, and stimulate oxidation reactions (Fig. 3a). At each experiment, 0.1 g of photocatalyst was mixed with 100 mL of LIND of concentration 0.8 mg/L. The second setup (Fig. 3b) consists of a glass reactor of 300 mL and a UVA lamp emitting at 375 nm at a power of 6 W. The UVA lamp is placed in a glass tubular housing, inserted at the center of the reactor whereas an air pump injects air. At each experiment, 1 g/L of photocatalyst was mixed with 100 or 200 mL of LIND solution of concentration 0.8 mg/L. In both setups, the suspensions were stirred mildly. Prior to UV irradiation, each suspension was kept in dark for 60 min until establishing equilibrium between adsorbed and bulk LIND.

Except for the synergistic adsorption and photocatalysis on the various TiO_2_/AC composites, the following processes were also investigated: (i) adsorption onto TiO_2_/AC-0.1 composite; (ii) photolysis without the use of adsorbent, catalyst or TiO_2_/AC composite; (iii) photocatalysis with bare TiO_2_. During the experiments, samples were collected, filtered with 0.2-μm syringe filters, centrifuged at 8000 rpm for 10 min in the case of the existence of TiO_2_ catalyst, extracted, and analyzed with GC-ECD. The photocatalytic activity of the composite materials TiO_2_/AC was investigated by measuring the total organic carbon (TOC) in liquid phase with a TOC analyzer (multi N/C 2100S Analytic Jena GmbH), and recoding the oxidation–reduction (redox) potential (ORP) and pH during the photocatalytic degradation with Vernier sensors.

### Mass transfer multi-compartment model

The dynamics of sorption on the porous particles of adsorbents can be modeled taking into account the following four mass-transfer processes (Stavrinou et al. 2023a, b): (i) the mass-transfer from the bulk to the external surface of particles, which is governed by the film diffusion coefficient, ${k}_{f}$; (ii) the sorption on the external surface of particles; (iii) the mass-transfer inside the pore space of the particles through molecular diffusion in macro-/meso-pores, and surface diffusion in micro-pores, governed by the effective pore, ${D}_{e}$, and surface, ${D}_{s}$, diffusion coefficients, respectively; (iv) the sorption on the pores of the particles. With the assumption that the LIND sorption on the pore-walls of adsorbent is a reversible linear and instantaneous process with equilibrium constant, ${K}_{e}$, the mass balances of dissolved and adsorbed LIND concentrations can be converted into a set of particle-averaged ordinary differential equations (ODEs) (Goto et al. 1990; Lesage et al. 2010), depending on the mass-transfer coefficients, ${k}_{M}$, and ${k}_{m}$, for macro-/meso-pores and micro-pores, respectively. The transient responses of LIND concentration in bulk, ${C}_{b}$, mean LIND concentration in macropores, $\overline{{C }_{M}}$, mean LIND concentration adsorbed on meso-/macro-pores, $\overline{{Q }_{M}}$, mean LIND concentration adsorbed on micro-pores, $\overline{{Q }_{m}}$, and LIND concentration adsorbed on external grain surface, $\overline{{Q }_{f}}$, are given by the following equations:

4 $$\frac{d{C}_{b}}{dt}={a}_{s}\left(\frac{\omega -1}{\omega }\right)\left[{k}_{M}\left({C}_{b}-\overline{{C }_{M}}\right)+{k}_{m}\left({C}_{b}-\frac{\overline{{Q }_{m}}}{{K}_{e}}\right)+{k}_{f}\left({C}_{b}-\frac{\overline{{Q }_{f}}}{{K}_{e}}\right)\right]$$

5 $$\frac{d\overline{{Q }_{M}}}{dt}=\frac{{a}_{s}{k}_{M}{K}_{e}}{{\varepsilon }_{M}+{S}_{M}{\rho }_{g}{K}_{e}}\left({C}_{b}-\overline{{C }_{M}}\right)$$

6 $$\frac{d\overline{{Q }_{m}}}{dt}=\frac{{a}_{s}{k}_{m}{K}_{e}}{{S}_{m}{\rho }_{g}}\left({C}_{b}-\overline{\frac{{Q }_{m}}{{K}_{e}}}\right)$$

7 $$\frac{d{Q}_{f}}{dt}=\frac{{a}_{s}{k}_{f}}{{S}_{f}{\rho }_{g}}\left({C}_{b}-\frac{{Q}_{f}}{{K}_{e}}\right)$$

The contribution of each compartment to the total sorption of LIND is given by the following quantities:

8a $${q}_{M}=\overline{{Q }_{M}}{S}_{M}$$

8b $${q}_{m}=\overline{{Q }_{m}}{S}_{m}$$

8c $${q}_{f}={Q}_{f}{S}_{f}$$

where ${S}_{M}$, ${S}_{m}$, and ${S}_{f}$ are the specific surface areas for maco-/meso-porosity, micro-porosity, and external surface of the particles, respectively. The total adsorbed mass, ${q}_{t}$, is obtained with a mass balance over the various compartments of the particle, including the dissolved LIND remaining in macropores, namely:

9 $${q}_{t}={q}_{M}+{q}_{m}+{q}_{f}+{V}_{M}\overline{{C }_{M}}$$

Once the total specific surface area, ${S}_{BET}$, has been calculated from N_2_ sorption, the specific surface area, ${S}_{M}$, and pore volume, ${V}_{M}$, for macro-/meso-porosity can be estimated from MIP data (${S}_{\text{MIP}}$ and ${V}_{\text{MIP}}$). Additionally, the specific surface area for micro-pores, ${S}_{m}$, can be approximated by:

10 $${S}_{m}={S}_{\text{BET}}-{S}_{M}$$

The external specific surface area, ${a}_{s}$, of spherical particles with their radii, ${r}_{g}$, following a number-based distribution function, ${f}_{N}\left({r}_{g}\right)$, is given by:

11 $$a_s={{\int_0^\infty}}f_N\left(r_g\right)\left(\frac3{r_g}\right)dr_g$$

and the external surface area of particles, ${S}_{f}$, is estimated approximately by the relationship:

12 $${S}_{f}=\frac{{a}_{s}}{{\rho }_{g}}$$

where ${\rho }_{g}$ is the bulk density of porous particles. The macro-/meso-porosity, ${\varepsilon }_{M}$, and the fraction of the bulk liquid volume, $\omega$, are defined by:

13 $${\varepsilon }_{M}={\rho }_{g}{V}_{M}$$

14 $$\omega =\frac{1}{1+{m}_{V}{\rho }_{g}}$$

respectively, where ${m}_{V}$ is the concentration of particles in liquid phase.

Equations (4), (5), (6), and (7) are solved repeatedly using the initial condition: ${C}_{b}={C}_{b0}$, $\overline{{C }_{M}}=\overline{{Q }_{M}}=\overline{{Q }_{m}}=\overline{{Q }_{f}}=0,$ at $t=0$, so that the predicted transient responses of the LIND concentration in the bulk, ${C}_{b}\left(t\right)$, match the experimental data from kinetic sorption tests. This allows for the estimation of parameter values: ${K}_{e},{k}_{M},{k}_{m},{k}_{f}$. Additionally, the corresponding values of effective pore, ${D}_{e}$, and surface, ${D}_{s}$, diffusion coefficients can be determined by using the following relationships (Lesage et al. 2010):

15 $${D}_{e}=\frac{{r}_{P}}{5\left(\frac{1}{{k}_{M}}-\frac{1}{{k}_{f}}\right)}$$

16 $${D}_{s}=\frac{{r}_{P}}{5\left(\frac{1}{{k}_{m}}-\frac{1}{{k}_{f}}\right)}$$

### Sorption and photocatalysis model

Before activating the UV-light, the sorption of dissolved LIND onto the surface of TiO_2_ or TiO_2_/AC composite materials can be regarded as a reversible process consisting of adsorption and desorption steps, described by the Langmuir kinetic model, namely:

17 $$\frac{dS}{dt}={k}_{a}C\left({q}_{\text{max}}-q\right)-{k}_{d}q$$

18 $${C}_{b}+q\left(W/{V}_{L}\right)={C}_{b0}$$

where ${q}_{\text{max}}$ is the maximum LIND sorption capacity of the material (g-mol/kg), $W$ is the catalyst mass (kg), ${V}_{L}$ is the solution volume (m^3^), and ${k}_{a}$ (m^3^ kg^−1^ s^−1^), ${k}_{d}$ (s^−1^) are the kinetic constants of adsorption and desorption, respectively, interrelated by:

19 $${K}_{L}=\frac{{k}_{a}}{{k}_{d}}$$

The maximum sorption capacity of each material was calculated approximately and fixed by using the relationship:

20 $${q}_{\text{max}}=\frac{{S}_{A}}{{{D}_{\text{mol}}}^{2}{N}_{A}}$$

where ${S}_{A}$ is the specific surface area (m^2^/kg), ${N}_{A}$ (= 6.023 × 10^23^ molecules/g-mol) is the Avogadro number, and ${D}_{\text{mol}}$ is the diameter of LIND molecule (~ 0.6 nm, Sprynskyy et al. 2007). When switching on the UV-light, the simultaneous sorption and photodegradation of LIND on solid grains is described by the mass balances:

21 $$\frac{dC}{dt}=-\left(\frac{W}{{V}_{L}}\right)\left[{k}_{a}C\left({q}_{\text{max}}-q\right)-{k}_{d}q\right]$$

22 $$\frac{dS}{dt}={k}_{a}C\left({q}_{\text{max}}-q\right)-{k}_{d}q-{k}_{r}q$$

where ${k}_{r}$ is the pseudo-first order surface reaction constant. The initial concentrations, ${C}_{b}={C}_{L0}$ and $q={q}_{0}$, are those measured at the end of LIND sorption in darkness.

## Results and discussion

### Physicochemical characterization

#### Ν_2_ sorption isotherms and mercury intrusion porosimetry

**Fig. 4:**
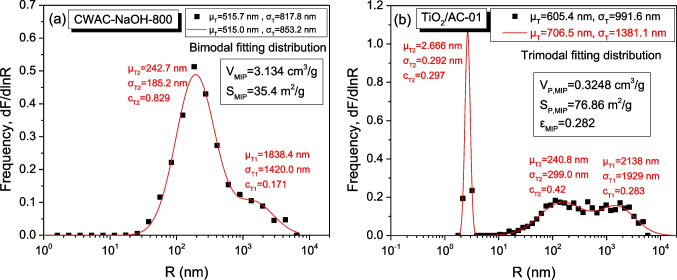
Throat radius distribution obtained from Hg intrusion curve for **a** CWAC-NaOH-800, **b** TiO_2_/AC-0.1. The mean value, μ, standard deviation, σ, and contribution fraction, c, of the total and component size distributions are provided

**Fig. 5:**
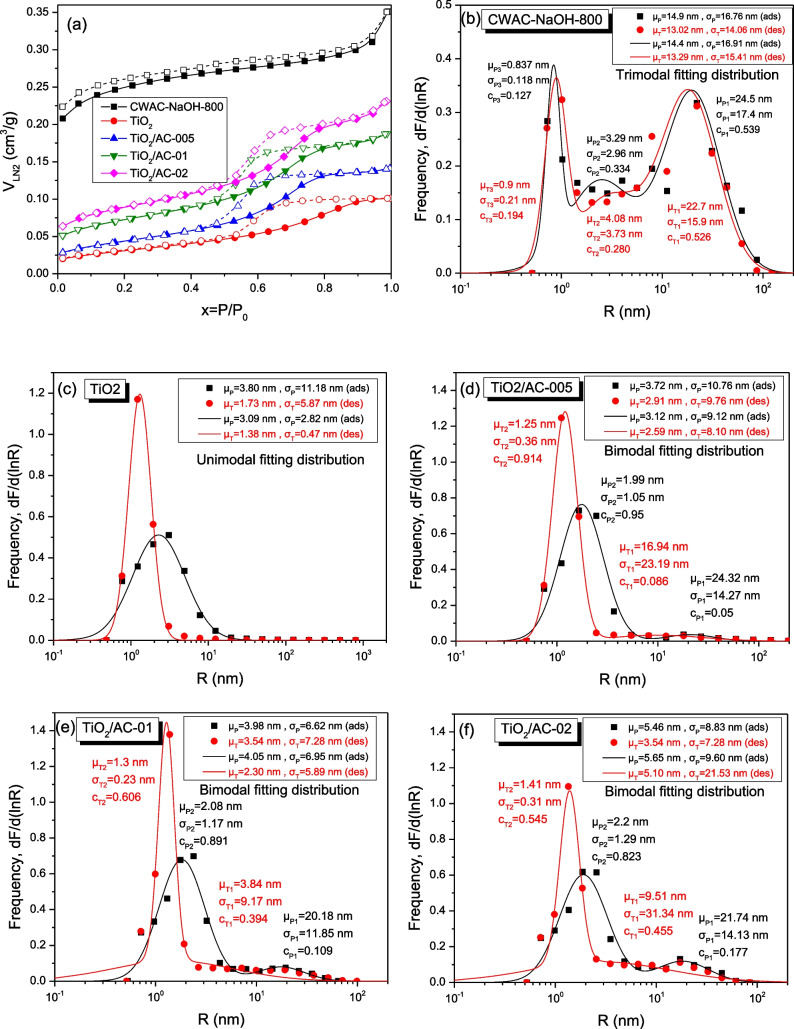
**a** N_2_ sorption isotherms of synthesized porous materials. Pore (P) and throat (T) radius distributions obtained from N_2_ adsorption/desorption isotherms of **b** CWAC-NaOH-800, **c** TiO_2_, **d** TiO_2_/AC-0.05, **e** TiO_2_/AC-0.1, **f** TiO_2_/AC-0.2. The mean value, μ, standard deviation, σ, and contribution fraction, c, of the total and component distributions are provided

**Fig. 6:**
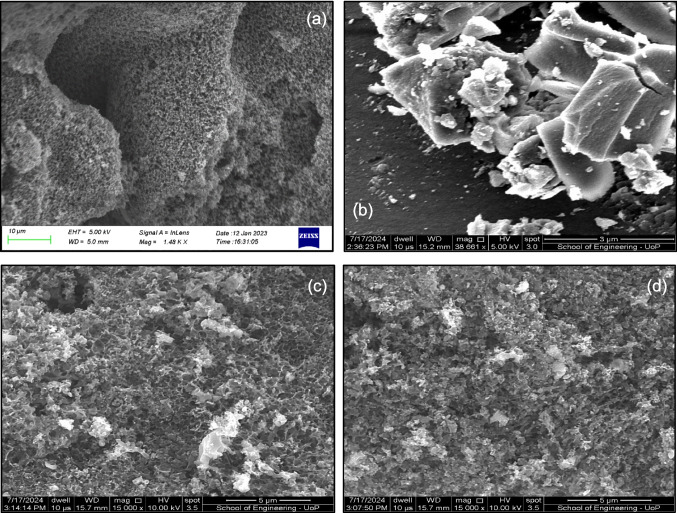
SEM images of the adsorbents: **a** CWAC-NaOH-800, **b** TiO_2_, **c** TiO_2_/AC-01, **d** TiO_2_/AC-02

On the full throat-radius distribution of AC (CWAC-NaOH-800), obtained from differentiation of the mercury intrusion curve (Fig. 4a), it is evident that the porosity is dominated mostly by macro-pores (*R* > 50 nm), and secondarily by meso-pores (2 nm < *R* < 50 nm), while the micro-pores (*R* < 2 nm) are not visible. However, the micro-pores become visible in the corresponding throat radius distribution of the composite adsorbent TiO_2_/AC-0.1 (Fig. 4b), because of the presence of TiO_2_ particles. The N_2_ adsorption–desorption isotherms of all porous materials are shown in Fig. 5a; the pore-size distribution, obtained from the adsorption branch, and the throat size distribution, obtained from the desorption branch, are shown in Fig. 5b–f, whereas the specific surface area, ${S}_{BET}$, total pore volume, ${V}_{L,N2}$, are given in Table 1. The degree of hysteresis between the two branches of N_2_ sorption isotherms is negligible for CWAC-NaOH-800 while the hysteresis widens, and the pore volume decreases (Table 1) with the percentage of AC mass decreasing and TiO_2_ mass increasing (Fig. 5a). This means that the meso- and micro-pores of AC resemble parallel capillary tubes (holes) penetrating inside the AC grains (Fig. 6a–SEM images), where the pore sizes are comparable to the throat sizes (Fig. 5b). However, with the incorporation of TiO_2_ nanoparticles (Fig. 5c) in the pore space of AC, pore network effects become evident by creating a micro-porous network with pore sizes higher than throat sizes (Fig. 5d–f). In the microporous network, created by the TiO_2_ nanoparticles aggregation, the pore radii range from 2.0 to 3.0 nm, and the throat radii range from 1.2 to 1.4 nm (T2 component distribution, Fig. 5c–f). In the TiO_2_/AC mesoporous network, the pore radii (P1) range from 20.0 to 25 nm (P1 component distribution), while the throat radii (T1) decrease from 22.7 to 4–17 nm (T1 component distribution) as the mass fraction of TiO_2_ increases (Fig. 5b, d–f). It is observed that the SBET surface area and VLN2 total pore volume of TiO_2_/AC composites decrease with the AC mass decreasing, as the contribution fraction of AC to the overall material porosity weakens (Table 1).

**Table 1:** Pore space properties of the materials

Adsorbent/photocatalyst	${S}_{BET}$(m^2^/g)	${V}_{L,N2}$(cm^3^/g)
CWAC-NaOH-800	694.1	0.351
TiO_2_	71.4	0.101
TiO_2_/AC-0.2	185.6	0.231
TiO_2_/AC-0.1	161.6	0.187
TiO_2_/AC-0.05	102.3	0.141

### Scanning electron microscopy images of the adsorbents/photocatalysts

The SEM images of the CWAC-NaOH-800, TiO_2_ nanoparticles and TiO_2_/AC-0.1 and TiO_2_/AC-0.2 composites are presented in Fig. 6a–d. As shown in Fig. 6a, the CWAC-NaOH-800 is dominated by meso- and macro-pores (Stavrinou et al. 2023b). On the other hand, TiO_2_ displays a coarse surface dominated by aggregated particles (Fig. 6b). When the aggregates of TiO_2_ nanoparticles are incorporated into the AC structure (TiO_2_/AC-0.1, Fig. 6c and TiO_2_/AC-0.2, Fig. 6d), they might block or be inserted into the macro-pores of AC by reducing the total pore volume (Table 1). Similar results have been reported elsewhere (Wang et al. 2007; Xue et al. 2011).

### X-ray diffraction analysis of adsorbents/photocatalysts

**Fig. 7:**
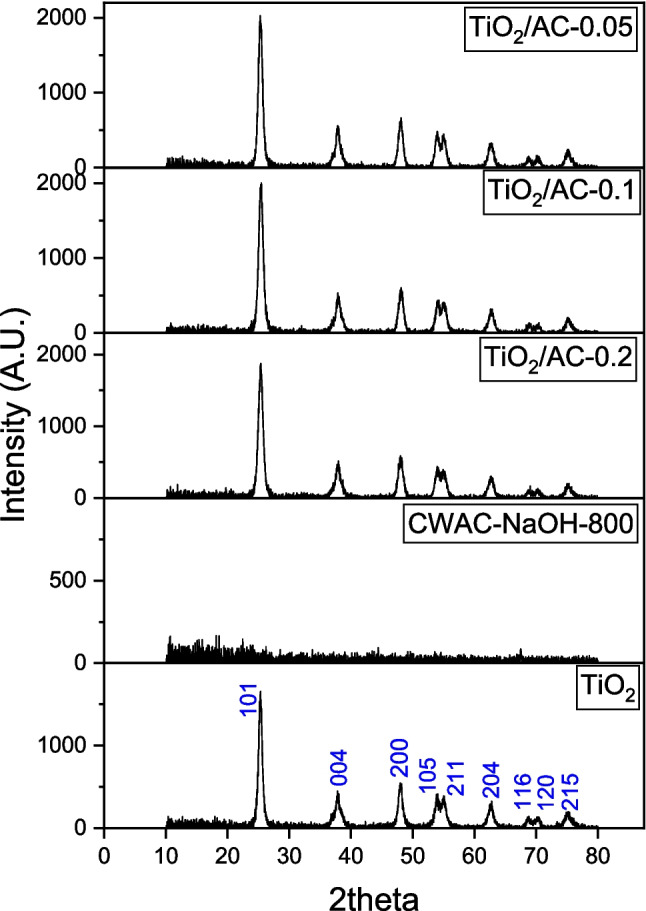
X-ray diffraction patterns of the materials CWAC-NaOH-800, TiO_2_, TiO_2_/AC-0.2, and TiO_2_/AC-0.1, TiO_2_/AC-0.05

The XRD patterns of the materials CWAC-NaOH-800, TiO_2_, TiO_2_/AC-0.2, TiO_2_/AC-0.1, and TiO_2_/AC-0.05 are presented in Fig. 7, and the corresponding estimated size of crystallites, $D$ (Eq. (1), is shown in Table 2. The XRD pattern of the activated carbon CWAC-NaOH-800 is indicative of its amorphous nature. On the other hand, TiO_2_ and TiO_2_/AC composites are highly crystalline with the anatase phase being predominant on the XRD spectrum. This is revealed by the presence of six main diffraction peaks at about 25.3°, 37.8°, 48.0°, 53.8°, 55.1°, and 62.8°, corresponding to (101), (004), (200), (105), (211), and (204) planes of anatase. The absence of rutile crystalline phase in the TiO_2_/AC composites may be due to the calcination temperature of 450 °C, as the anatase phase tends to be the stable form at lower temperatures (Wang et al. 2016). In addition, the high surface area of AC matrix can inhibit the conversion of anatase to rutile due to the large interfacial energy, which creates anti-calcination effects (Li et al. 2007). The crystallite sizes of TiO_2_ and TiO_2_/AC composites are comparable (Table 2), but the $D$ size has the tendency to increase with the surface area, ${S}_{BET}$ decreasing (Table 1), which may be related to the more coarse grains and particle agglomeration, indicated also from SEM images (Fig. 6b–d) (Amorós-Pérez et al. 2021). In addition, the smaller crystallite sizes of TiO_2_/AC composites might be attributed to the decomposition products of the AC into the crystalline structure of TiO_2_ (Martins et al. 2017).

**Table 2:** Crystallite size of TiO_2_ and TiO_2_/AC composites

Adsorbent/photocatalyst	*D* (nm)
TiO_2_	13.9
TiO_2_/AC-0.2	9.9
TiO_2_/AC-0.1	10.7
TiO_2_/AC-0.05	10.8

### Thermogravimetric analysis of adsorbents/photocatalysts

**Fig. 8:**
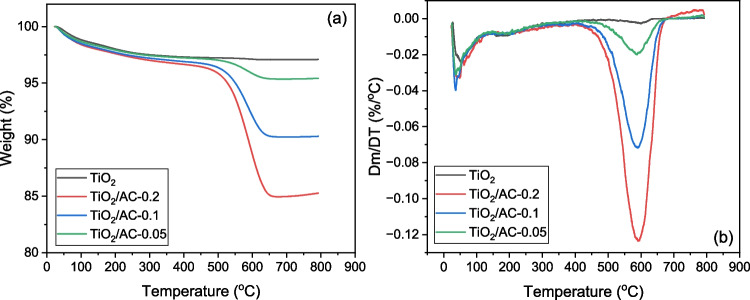
**a** Thermogravimetric (TG), **b** differential thermogravimetric (DTG) curves of the materials TiO_2_, TiO_2_/AC-0.2, TiO_2_/AC-0.1, TiO_2_/AC-0.05

The weight loss evolution of the samples during calcination is determined with thermogravimetric analysis. The thermogravimetric (TG) and differential thermogravimetric (DTG) curves of TiO_2_ and TiO_2_/AC composites are shown in Fig. 8. From the construction of DTG curves, the precise location of the weight loss temperatures can be obtained. Two main weight losses are observed, over 20–80 °C and 450–650 °C, for all samples (Fig. 8b). The first corresponds to the escape of adsorbed water, and the second is attributed to the combustion of carbon in the air. After the temperature of 650 °C, there are no mass losses, and the remaining mass contains TiO_2_ and AC ash. It is obvious that with the carbon content increasing there is an increase in the mass loss (Fig. 8a). Finally, the carbon content (%) of each sample was calculated by subtracting the main weight loss over the band 450–650 °C, centered at 550 °C, and it was found equal to 11.99%, 6.73%, and 1.73% for TiO_2_/AC-0.2, TiO_2_/AC-0.1, and TiO_2_/AC-0.05, respectively (Wang et al. 2007; Amorós-Pérez et al. 2021).

### Adsorption of LIND onto the adsorbent CWAC-NaOH-800

#### Effect of the initial pH and contact time on the adsorption capacity of CWAC-NaOH-800

**Fig. 9:**
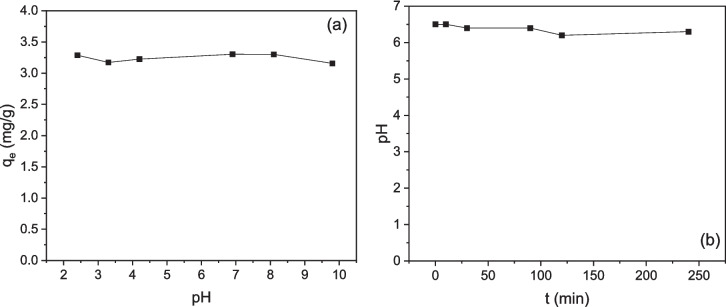
**a** Effect of pH on LIND adsorption onto CWAC-NaOH-800 (C_0_ = 0.8 mg/L, contact time = 120 min, adsorbent dosage = 0.2 g/L), **b** variation of pH during the adsorption of LIND onto CWAC-NaOH-800 (C_0_ = 3.1 mg/L, pH_0_ = 6.5, adsorbent dosage = 0.2 g/L)

The effect of pH on LIND adsorption onto CWAC-NaOH-800 is shown in Fig. 9a. Evidently, the adsorption capacity, ${q}_{e}$ (mg/g), does not change with the increase of pH from 2 to 10. When measuring the ζ-potential of CWAC-NaOH-800 in previous work (Stavrinou et al. 2023b), the surface charge of the adsorbent was found insensitive to the pH over the range 2–10, and no isoelectric point was identified, indicating the lack of any preference of CWAC-NaOH-800 to adsorb LIND molecules under acidic or alkaline conditions. In addition, the nonpolar nature of LIND (Brown 2000) reduces the possibility of electrostatic interactions with the charged surface of the adsorbent. Furthermore, the pH was measured during adsorption without observing any change of it, confirming the aforementioned conclusion (Fig. 9b). The insensitivity of the AC sorption capacity to pH, with respect to LIND has been reported earlier as well (Elkady et al. 2013; Tor et al. 2013).

**Fig. 10:**
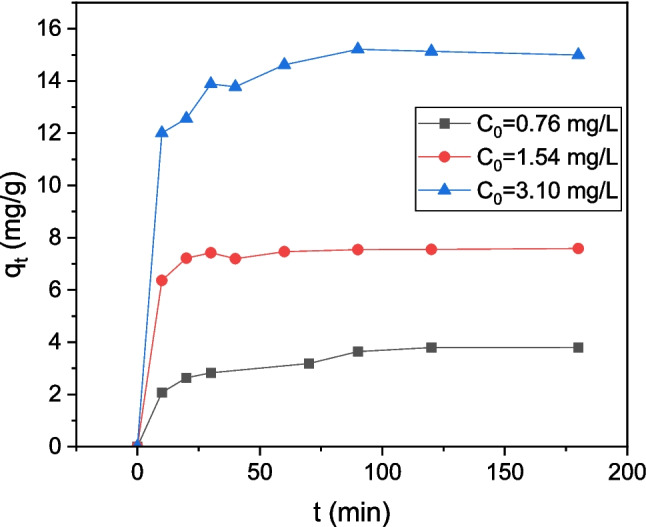
**a** Effect of contact time on LIND adsorption onto CWAC-NaOH-800 (C_0_ = 0.8 mg/L, pH = 6.9, adsorbent dosage = 0.2 g/L)

The effect of the treatment time on LIND adsorption onto CWAC-NaOH-800 at three initial LIND concentrations (${C}_{0}$=0.76, 1.54, 3.10 mg/L) is presented in Fig. 10. In the first 30 min, the adsorption capacity of CWAC-NaOH-800 was increased rapidly, most likely due to the abundance of the available adsorption sites on the surface of CWAC-NaOH-800. Afterwards the amount of adsorbed LIND was increased slowly, probably because of the diffusion into the pores of the adsorbent and gradual occupation of vacant sites (Moradnejadi et al. 2019). Finally, the equilibrium was established after 120 min, which was the duration of all experiments.

### Simulation of LIND sorption dynamics with the multi-compartment model

**Fig. 11:**
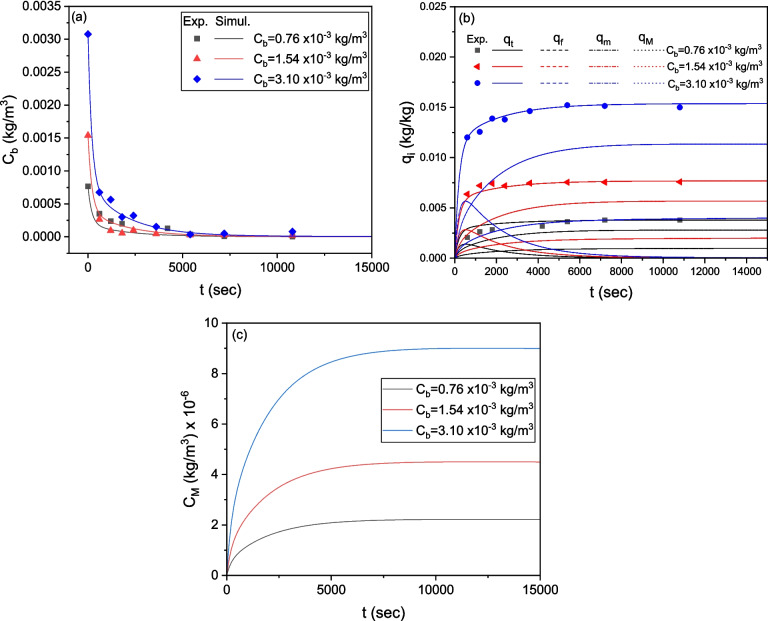
Comparison of experimental measurements with numerical predictions of the multi-compartment model for CWAC-NaOH-800. **a** Transient response of LIND concentration in bulk liquid. **b** Transient response of the total concentration of adsorbed LIND and its distribution into each compartment of adsorbent. **c** Transient response of the mean LIND concentration in meso-/macro-pores of adsorbent

To increase the accuracy of model predictions, it is essential to approximate the average particle size of the granular CWAC-NaOH-800. The grain size distribution, based on mass (Table 3) (Stavrinou et al. 2023b), was converted into a number-based distribution which was then utilized to calculate the mean value, standard deviation, and external surface area, ${a}_{s}$, of AC particles using Eq. (11) (Table 4). To conduct kinetic sorption tests, three different initial concentrations of LIND (0.76, 1.54, 3.10 mg/L) were employed. The ODEs (Eqs. 4, 5, 6, and 7) were solved using central finite differences while the Bayesian estimator of Athena Visual Studio 14 was used to fit with inverse modeling the numerical solution to the experimental datasets (Stewart and Caracotsios 2008), simultaneously for the three transient responses of the LIND concentration obtained from the three tests. The estimated values of the linear equilibrium constant for adsorption, ${K}_{e}$, and the mass-transfer coefficients ${k}_{M},{k}_{m}, {k}_{f}$ and the post-calculated effective pore, ${D}_{e}$, and surface, ${D}_{s}$, diffusion coefficient (Eqs. 15 and 16, respectively) are presented in Table 5. The value of ${D}_{e}$ (7.368 × 10^−10^ m^2^/s) is of the same order of magnitude with the molecular diffusion coefficient of (i) LIND in water (${D}_{m}=$ 4.98 × 10^−10^ m^2^/s; Jury et al. (1983)) and (ii) pesticides in water (${D}_{m}=$ 7.3 × 10^−10^ m^2^/s; Sarraute et al. (2019)). The simulated transient responses are compared with the experimental results of kinetic tests in Fig. 11a, b. The contribution of each compartment (meso-/macro-porous region, micro-porous region, and external surface) to the total sorption is depicted in Fig. 11b, whereas the predicted concentration of LIND entrapped in the macro-porosity is presented in Fig. 11c.

**Table 3:** Mass-based grain size distribution of CWAC-NaOH-800

Granule size range (μm)	Mass percentage (%)
50–125	5.08
125–250	26.13
250–500	46.04
500–1000	23.46

**Table 4:** Input parameters in the multi-compartment model for CWAC-NaOH-800

$\langle {r}_{g}\rangle$(μm)	${\sigma }_{g}$(μm)	${a}_{s}$(m^−1^)	${\rho }_{g}$(kg/m^3^)	${m}_{V}$(kg/m^3^)	${V}_{M}$(m^3^/kg)	${S}_{M}$(m^2^/kg)	${S}_{m}$(m^2^/kg)	${S}_{f}$(m^2^/kg)	${C}_{b0}$(kg/m^3^)
71	44.3	53,007	260.0	0.2	3.134 × 10^−3^	35.4 × 10^3^	641.1 × 10^3^	203.87	0.76 × 10^−3^ 1.54 × 10^−3^ 3.10 × 10^−3^
$2{r}_{g}$(μm)	87.5	187.5	375	750					
${f}_{N}$	0.6079	0.3178	0.0700	0.0043

**Table 5:** Estimated mass transfer coefficients by the multi-compartment model for CWAC-NaOH-800

${K}_{e}$(m)	${k}_{M}$(m s^−1^)	${k}_{m}$(m s^−1^)	${k}_{f}$(m s^−1^)	${D}_{e}$(m^2^ s^−1^)	${D}_{s}$(m^2^ s^−1^)
3.558 × 10^−2^	2.708 × 10^−5^	8.992 × 10^−6^	5.719 × 10^−5^	7.368 × 10^−10^	1.46 × 10^−10^
Sum of squares of residuals	1.382 × 10^−7^

The multi-compartment model predicts satisfactorily the sorption kinetics for all initial LIND concentrations and treatment times, with the best fit occurring at the highest initial concentration (${C}_{0}$=3.10 mg/L) (Fig. 11a, b). At early times, LIND diffuses very fast toward the external surface of particles, and is adsorbed onto it (Fig. 11b). As the LIND concentration in bulk decreases rapidly, the adsorbed concentration on the external particle surface also decreases since it is at equilibrium with its concentration in the bulk. Gradually, LIND is transferred via molecular diffusion and adsorbed on the meso-/macro-pores, while surface diffusion contributes to the transfer and sorption of LIND molecules on the micro-pores (Fig. 11b). The contribution fraction of micro-pore surface diffusion, ${q}_{m}$ (mg/g) to the total sorption is lower than that of macro-/meso-pore molecular diffusion, ${q}_{M}$, mainly due to the higher percentage of pores with sizes in the meso-/macro-porous range. The average concentration of LIND contained in the meso-/macro-pores is quite low (Fig. 11c) and has a negligible effect on the total adsorption capacity, ${q}_{t}$.

### Effect of the initial concentration of LIND on the adsorption capacity of CWAC-NaOH-800

**Fig. 12:**
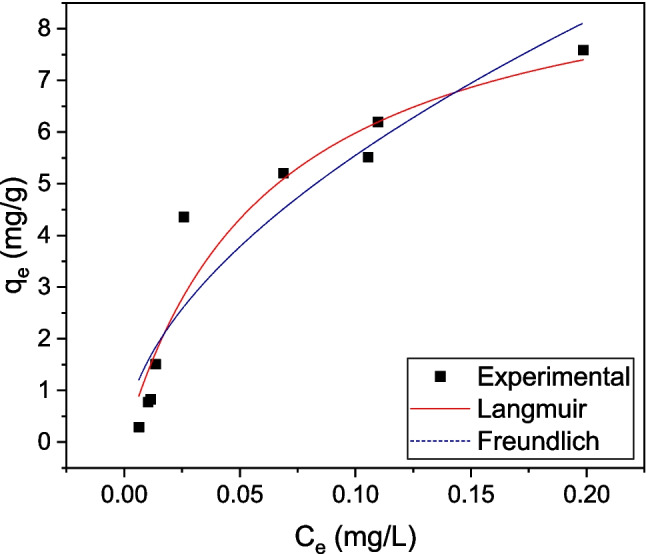
Effect of the initial LIND concentration on its adsorption onto CWAC-NaOH-800 (contact time = 120 min, pH = 6.9, adsorbent dosage = 1 g/L) and fitting the isotherm with Langmuir and Freundlich models

The experimentally measured isotherm for LIND over a concentration range 0.1–5 mg/L was fitted with Langmuir (Langmuir 1918) and Freundlich (Freundlich 2017) models (Fig. 12).

Langmuir isotherm:

23 $${q}_{e}=\frac{{q}_{\text{max}}{K}_{L}{C}_{e}}{1+{K}_{L}{C}_{e}}$$

where ${q}_{\text{max}}$ (mg/g) is the maximum adsorption capacity of the adsorbent for monolayer coverage and ${K}_{L}$ (L/mg) is the Langmuir adsorption constant related to the affinity of binding sites and the free energy of adsorption. There is the hypothesis that the Langmuir adsorption capacity may be correlated with the specific surface area and porosity of the adsorbent, implying that larger surface areas and pore volumes result in a higher adsorption capacity (Mehmeti et al. 2022).

Freundlich isotherm:

24 $${q}_{e}={K}_{F}{C}_{e}^{1/n}$$

where ${K}_{F}$ ((mg/g)(L/mg)^1/n^) is the Freundlich adsorption constant related to the maximum adsorption capacity of the adsorbent and intensity of adsorption, and varying with the heterogeneity of its surface. The Freundlich isotherm describes multilayer adsorption where interactions between adsorbed molecules take place (Aggelopoulos et al. 2017).

The correlation coefficient of the Langmuir isotherm (${R}^{2}$=0.94) is higher than that calculated by Freundlich isotherm (${R}^{2}$=0.90), and Langmuir isotherm appears to interpret better the LIND sorption onto CWAC-NaOH-800 at equilibrium (Table 6). The maximum sorption capacity was estimated to be equal to 9.74 mg/g, which is considered a satisfactory value (Table 6) with reference to earlier studies (Mehmeti et al. 2022). A possible explanation of why a model describing monolayer adsorption, like Langmuir model, fits better to the experimental results relies on the pore structure of the AC and the steric hindrance of pores which prevent the growth of additional layers of LIND molecules (Amrhar et al. 2021; Sellaoui et al. 2015).

**Table 6:** Isotherm parameters for the adsorption of LIND onto CWAC-NaOH-800

Langmuir			Freundlich		
${q}_{max}$ mg/g	${K}_{L}$ L/mg	${R}^{2}$	${K}_{F}$(mg/g)(L/mg)^1/n^	$1/n$	${R}^{2}$
9.74	15.93	0.94	19.86	0.55	0.90

The linear sorption constant estimated by the multi-compartment model (Table 5) is normalized with respect to the total surface area according to the following:

25 $${{K}_{e}}^{\prime}={K}_{e}\left({S}_{M}+{S}_{m}+{S}_{f}\right)$$

and it is obtained ${{K}_{e}}{\prime}$=20.08 $\text{L}/\text{mg}$, which is comparable to the Langmuir sorption constant (Table 6).

### Adsorption mechanism of LIND onto CWAC-NaOH-800

The non-polar nature of LIND plays a significant role on its adsorption on ACs. AC typically exhibits hydrophobic properties, namely it tends to repel water molecules and adsorbs preferentially non-polar or hydrophobic compounds. So the highly hydrophobic character of LIND suggests that the hydrophobic effect mainly governs its interaction with AC (Erro et al. 2023). Chlorine atoms may introduce a polarizability into the LIND molecule because they are more electronegative than carbon, and hence, a partial negative charge on the chlorine atoms and a partial positive charge on the surrounding carbon atoms may be produced. This polarizability may lead to weak dipole–dipole interactions contributing slightly to the adsorption process (Sprynskyy et al. 2008). However, the probability of the existence of strong electrostatic forces between the charged surface of the absorbent and LIND is very low given that the process would be pH-dependent, and the adsorbent would have an isoelectric point, but none of these two cases was realized (Fig. 9). According to earlier work investigating the adsorption of emerging pollutants on AC, the adsorption of hydrophobic organic compounds was not affected by pH while the adsorption of hydrophilic organic compounds was significantly affected by pH, and hence, electrostatic and specific sorbate-sorbent interactions between the pollutants and the AC surface might affect the adsorption of hydrophilic compounds (Jeirani et al. 2017). The formation of covalent bonds or other strong chemical interactions is not very likely since functional groups such as hydroxyl (-OH) or carboxyl (-COOH) should be present on the surface of the adsorbent CWAC-NaOH-800, but no such functional groups were detected in the ATR-FTIR spectrum, while Raman spectroscopy revealed the fully graphitic nature of CWAC-NaOH-800 (Stavrinou et al. 2023b). Summarizing, the dominant mechanism of LIND adsorption onto CWAC-NaOH-800 is the hydrophobic interaction due to the absence of polarity in both the adsorbent and adsorbate.

### Synergistic adsorption and photocatalysis of LIND

**Fig. 13:**
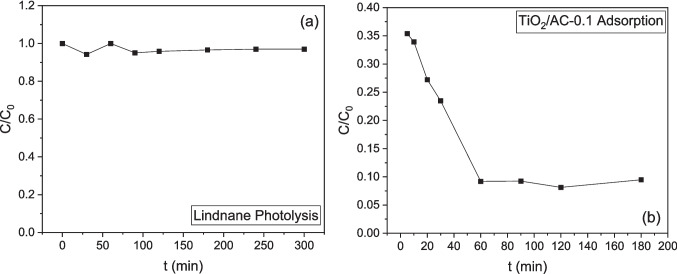
Kinetic experiments of LIND **a** photolysis, **b** adsorption onto TiO_2_/AC-0.1 (C_0_ = 0.8 mg/L, TiO_2_/AC-0.1 dosage = 0.1 g/L)

The kinetic experiments of LIND photolysis and adsorption onto TiO_2_/AC-0.1 composite at the setup of the UVA oven are presented in Fig. 13. The plots are interpreted in terms of the dimensionless LIND concentration $C/{C}_{0}$ versus time, where $C$ is the concentration at time t and ${C}_{0}$ is the initial concentration of LIND. According to the photolysis experiment, the LIND concentration did not change after 300 min, revealing that photodegradation did not occurred without the presence of a photocatalyst in the process (Fig. 13a). On the other hand, the composite TiO_2_/AC-0.1 shows a satisfactory removal efficiency of LIND, with half of the LIND mass being adsorbed during the first 15 min, and the equilibrium being established after 60 min. The corresponding adsorption capacity is equal to 3.6 mg/g, which is comparable to the corresponding one of CWAC-NaOH-800 (${q}_{t}$=3.8 mg/g) when starting from the same initial LIND concentration (${C}_{0}$=0.8 mg/L) and AC dosage (*m* = 0.2 g/L) (Fig. 10). Although the surface area and the pore volume of the composite are lower than those of AC (Table 1), the LIND sorption capacity of TiO_2_/AC-0.1 was not affected sensibly, probably due to the strong LIND sorption onto TiO_2_.

**Fig. 14:**
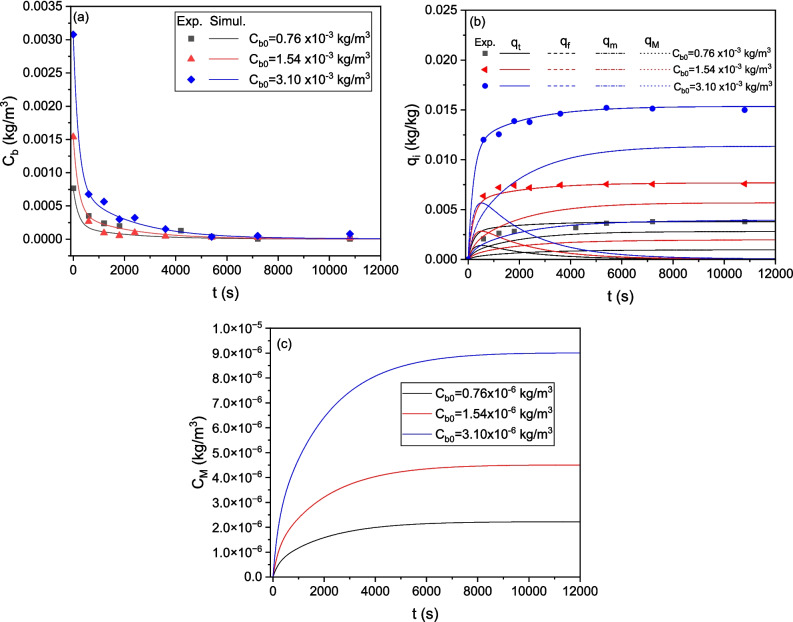
Comparison of experimental measurements with numerical predictions of the multi-compartment model for TiO_2_/AC-0.1 composite. **a** Transient response of LIND concentration in bulk liquid. **b** Transient response of the total concentration of adsorbed LIND and its distribution into each compartment of adsorbent. **c** Transient response of the mean LIND concentration in meso-/macro-pores of adsorbent

From the mass-based grain size distribution of TiO_2_/AC-0.1 (Table 7), the input parameters of the multicompartment model were calculated (Table 8), and the simultaneous inverse modeling of kinetic sorption tests at three initial LIND concentrations enabled the estimation of the relevant mass-transfer parameters (Table 9). The model fits satisfactorily to the experimental data for all initial concentrations and throughout the duration of experiments (Fig. 14a, b). As shown in Fig. 14b, during the initial stages of LIND adsorption, the contribution fraction of film diffusion to the sorption onto TiO_2_/AC-0.1 grains, ${q}_{f}$, is higher than that of molecular diffusion in meso-/macro-pores, ${q}_{\rm M}$. This is also reflected in the mass transfer coefficients ${k}_{M}, {k}_{f}$, where the film diffusion coefficient, ${k}_{f}$, is an order of magnitude higher than the mass transfer coefficient in the meso-/macro-pores, ${k}_{M}$ (Table 9). The surface diffusion in the micro-pores has a relatively weaker contribution fraction to sorption compared to the molecular diffusion in the meso-/macro-pores (Table 9, Fig. 14b), but significantly stronger than that of “pure” activated carbon (Fig. 11b), since the meso-/macro-porosity decreases with TiO_2_ mass fraction increasing (Fig. 5c–f). Likewise, the average concentration of LIND entrapped in the meso-/macro-pores of TiO_2_/AC-0.1 is quite low (Fig. 14c) and has a negligible effect on the total adsorption capacity, ${q}_{t}$.

**Table 7:** Mass-based grain size distribution of TiO_2_/AC-0.1

Granule size range (μm)	Mass percentage (%)
50–125	66.0
125–250	25.0
250–500	9.0
500–1000	0

**Table 8:** Input parameters in the multi-compartment model for TiO2/AC-0.1

$\langle {r}_{g}\rangle$(μm)	${\sigma }_{g}$(μm)	${a}_{s}$(m^−1^)	${\rho }_{g}$(kg/m^3^)	${m}_{V}$(kg/m^3^)	${V}_{M}$(m^3^/kg)	${S}_{M}$(m^2^/kg)	${S}_{m}$(m^2^/kg)	${S}_{f}$(m^2^/kg)	${C}_{b0}$(kg/m^3^)
45.84	22.14	67130	810.0	1	0.325 × 10^−3^	75.86 × 10^3^	161.3 × 10^3^	82.88	0.78 × 10^−3^ 1.41 × 10^−3^ 3.11 × 10^−3^
$2{r}_{g}$(μm)	87.5	187.5	375	750					
${f}_{N}$	0.961	0.037	0.0016	0

**Table 9:** Estimated mass transfer coefficients by the multi-compartment model for TiO_2_/AC-0.1

${K}_{e}$(m)	${k}_{M}$(m s^−1^)	${k}_{m}$(m s^−1^)	${k}_{f}$(m s^−1^)	${D}_{e}$(m^2^ s^−1^)	${D}_{s}$(m^2^ s^−1^)
2.66 × 10^−2^	2.362 × 10^−6^	1.552 × 10^−6^	1.84 × 10^−5^	0.84 × 10^−10^	1.32 × 10^−10^
Sum of squares of residuals	1.892 × 10^−7^

**Fig. 15:**
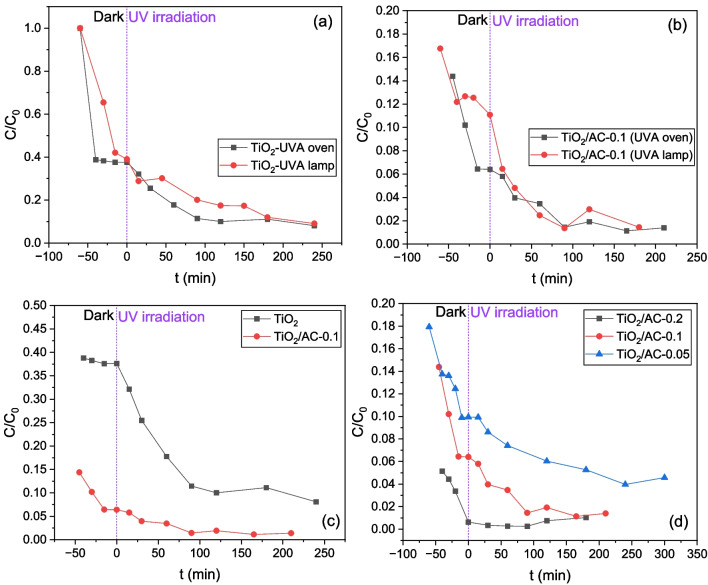
Transient responses of LIND concentration for kinetic tests of LIND adsorption and photocatalysis on: **a** bare TiO_2_ in UVA oven (22 W) and UVA lamp (6 W) setups; **b** TiO_2_/AC-0.1 composite in UVA oven and UVA lamp setups; **c** bare TiO_2_ and TiO_2_/AC composites in UVA oven setup; **d** TiO_2_/AC-0.2, TiO_2_/AC-0.1, TiO_2_/AC-0.05 composites in UVA oven setup (C_0_ = 0.8 mg/L, TiO_2_/AC dose = 0.1 g/L)

The synergistic adsorption-photocatalysis on TiO_2_ nanoparticles and TiO_2_/AC materials for the two experimental setups (UVA oven-22 W, UVA lamp-6 W) is shown in Fig. 15. TiO_2_ acts as an adsorbent in dark conditions and shows satisfactory photocatalytic removal of LIND under UV irradiation (Fig. 15a). The TiO_2_/AC composite presents much higher LIND sorption capacity than bare TiO_2_ (LIND removal efficiency 93.7% vs. 62.5%, Fig. 15b, c), which is also reflected in the overall performance of photocatalysts after having activated the UV-radiation (LIND removal efficiency 98.6% vs. 91.0%, Fig. 15c). In spite of the lower TiO_2_ mass in TiO_2_/AC-0.1 composite with regard to pure TiO_2_, the photodegradation efficiency is comparable for both catalysts (~ 78%, Fig. 15c). This might be explained by the co-adsorption mechanism where the co-existence of TiO_2_ and AC weakens the rate of electron/hole pair recombination which reduces the quantum yield of the TiO_2_ photocatalysis process, and is the main limitation to photocatalytic efficiency. The increase of the photocatalytic efficiency might be attributed to the formation of a contact interface between the different solid phases, in which AC acts as an adsorption trap for the organic pollutant, which is then more efficiently transferred to the TiO_2_ surface where it is immediately photo-degraded (da Silva and Faria 2003). The efficiency of the two setups in both cases is comparable even if the power of UVA oven is almost four times that of UVA lamp. This indicates that the radial propagation of the light emitted by UVA lamp leads to its better absorption by the catalyst particles compared to the UVA-oven.

A comparison of the LIND degradation efficiency for the three composites TiO_2_/AC-0.2, TiO_2_/AC-0.1, and TiO_2_/AC-0.05 is shown in Fig. 15d. In general, an increase of the adsorbent dosage leads to a decrease of the bulk LIND concentration. In the case of the composite containing the largest AC dosage, i.e., TiO_2_/AC-0.2, the photocatalytic degradation of LIND during UV-irradiation is unclear, since the ratio of AC to TiO_2_ mass is very high (1:2) and probably the composite acts mostly as an adsorbent rather than as catalyst. On the other hand, a high AC dosage could prevent the illumination of catalyst surface and limit its capacity to absorb light (Amorós-Pérez et al. 2021). Also, the enhanced sorption capacity and reduced photocatalytic activity of TiO_2_/AC-0.2 are associated with the higher surface area and pore volume (Table 1) and lower crystallite size (Table 2), respectively, given that the photoactivity of anatase increases with its crystallite size (Wang et al. 2007).

### Evaluation of the photocatalytic ability of TiO_2_/AC composites

**Fig. 16:**
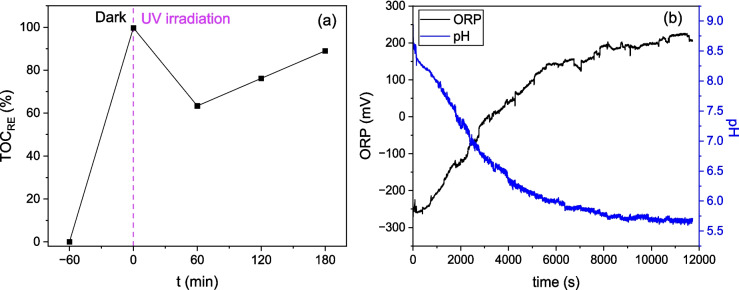
**a** TOC removal efficiency during the synergistic adsorption-photocatalysis with the TiO_2_/AC-0.1 composite. **b** Measurement of the oxidation–reduction potential (ORP) and pH during photocatalytic degradation with the TiO_2_/AC-0.05 composite (UVA lamp setup)

To confirm the photocatalytic activity of the TiO_2_/AC composites, the TOC was measured during the synergistic adsorption-photocatalysis process (Fig. 16a), while the oxidation reduction potential (ORP) and pH were recorded continuously during the photocatalysis process (Fig. 16b). The percentage of TOC removal efficiency, ${TOC}_{RE}$, was calculated by the following equation:

26 $${\text{TOC}}_{\text{RE}}=\frac{\text{TOC}\left(0\right)-\text{TOC}\left(t\right)}{\text{TOC}\left(0\right)}$$

where $TOC\left(0\right)$ is the initial TOC value (mg/L) and $TOC\left(t\right)$ is the TOC value at time *t* (mg/L).

The first TOC measurement was taken after 60 min of LIND adsorption on TiO_2_/AC-0.1 composite, before the UV lamp was turned on. As shown in Fig. 16a, up to that point a TOC removal efficiency equal to 99.7% was achieved. When the UV radiation was activated the TOC removal efficiency dropped to 63.4% after 60 min of adsorption-photocatalysis, probably due to photodegradation of adsorbed LIND and desorption of intermediate products from TiO_2_/AC-0.1 surface. Then, the TOC removal efficiency increased, and after 180 min of adsorption-photocatalysis reached ~ 89%, due to the degradation of harmful intermediates, confirming the regeneration of the composite during photocatalysis.

As shown in Fig. 16b, during photocatalysis, the pH tends to decrease, while the ORP tends to increase. The decrease in pH and increase in ORP indicate the production of hydroxyl radicals (·OH) leading to the oxidation of LIND. In general, the degradation of LIND is a complex process, where redox reactions constantly take place, such as chlorination, dechlorination, hydroxylation, hydrogenation, and dehydrogenation (Antonaraki et al. 2010). In the case of photocatalytic oxidation, the pollutant can either be directly oxidized by valence band holes or indirectly attacked by ·OH. Non-polar pollutants can be attacked indirectly by ·OH radicals. Since LIND is a hydrophobic/non-polar pollutant, oxidation is possible via hydroxylation of its ring. Hydroxylation is the prerequisite for ring cleavage, which directly generates the monocarboxylic or dicarboxylic acid products (Shah and Patel 2021).

### Inverse modeling of sorption-photocatalysis tests

**Fig. 17:**
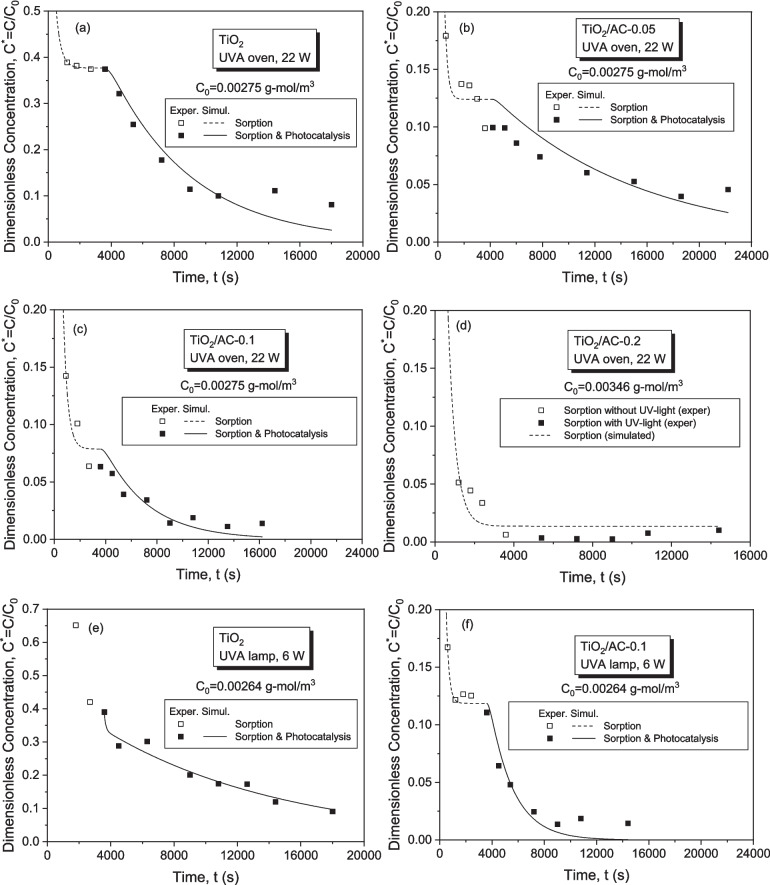
Comparison of experimental with simulated transient responses of the dimensionless concentration of LIND, $C/{C}_{0}$, for the systems **a** TiO_2_ in UVA oven, **b** TiO_2_/AC-0.05 in UVA oven, **c** TiO_2_/AC-0.1 in UVA oven, **d** TiO_2_/AC-0.2 in UVA oven, **e** TiO_2_ in UVA lamp, **f** TiO_2_/AC-0.1 in UVA lamp

The kinetic constants of LIND sorption on the various types of materials were estimated with inverse modeling of sorption data in darkness by using Eqs. (17) and (18). Then, the sorption parameter values were fixed, and the photo-degradation kinetic constant was estimated with inverse modeling of photocatalysis data by using Eqs. (21) and (22). For the inverse modeling, the Bayesian estimator of the Athena Visual Studio 14 was used, and the results are shown in Table 10 and Fig. 17a–f.

**Table 10:** Estimated parameter values of sorption and photocatalysis kinetics

Material	UVA light	${q}_{\text{max}}$(g-mol/kg)	${K}_{L}$(m^3^/kg)	${k}_{d}$×10^−4^ (s^−1^)	${k}_{r}$×10^−4^ (s^−1^)
TiO_2_	Oven, 22W	0.322	5.16 ± 0.21	12.14 ± 4.1	3.16 ± 1.13
TiO_2_/AC-0.05	Oven, 22W	0.462	15.38 ± 3.49	5.68 ± 2.55	1.01 ± 0.43
TiO_2_/AC-0.1	Oven, 22W	0.730	16.10 ± 12.12	2.30 ± 2.52	3.17 ± 1.34
TiO_2_/AC-0.2	Oven, 22W	0.838	87.16 ± 8.66	0.34 ± 0.37	-
TiO_2_	Lamp, 6W	0.322	6.26 ± 1.51	33.83	1.29 ± 0.52
TiO_2_/AC-0.1	Lamp, 6W	0.730	10.21 ± 1.20	5.70 ± 1.38	6.38 ± 3.17

It seems that the equilibrium sorption coefficient ${K}_{L}$ increases, and the desorption kinetic constant decreases, both profoundly, with the mass fraction of AC increasing (Table 10). This is also reflected in the gradually higher sorption capacity, ${q}_{\text{max}}$, of the materials as the percentage of AC in composite materials increases (Fig. 17a–d, Table 10). It is worth mentioning that the LIND sorption on TiO_2_/AC-0.2 composite is so strong that photodegradation is not “visible” with the LIND concentration in solution being stabilized at a low value very fast after having activated the UVA light (Fig. 17d). The estimated kinetic constant of photodegradation is of the same order of magnitude for all materials and is maximized when TiO_2_/AC-0.1 composite is used with the UVA lamp setup (Table 10, Fig. 17f). In addition, the model was unable to estimate low uncertainty parameter values from the sorption data in darkness for the system TiO_2_/UVA lamp (6 W), due to the limited number of measurements. In this case, all sorption and photocatalytic parameters were estimated simultaneously from measurements with the UVA lamp on (Fig. 17e).

From the comparison of the estimated kinetic constant, ${k}_{r}$, values for TiO_2_, TiO_2_/AC-0.1, and TiO_2_/AC-0.05 in the UVA oven (Fig. 17b–d), it seems that TiO_2_ and TiO_2_/AC-0.1 exhibit the maximum photocatalytic activity, which weakens for TiO_2_/AC-0.05 (${k}_{r}$=1.01 × 10^−4^ s^−1^), and is obscured by the high AC sorption capacity for TiO_2_/AC-0.2 (Table 10). Nevertheless, due to the strong sorption capacity of AC, the overall LIND removal efficiency for TiO_2_/AC-0.1 and TiO_2_/AC-0.05 composites is higher than that of TiO_2_ with corresponding final values of dimensionless LIND concentration $C/{C}_{0,\text{TiO}2/\text{AC}-0.1}$=0.014, $C/{C}_{0,\text{TiO}2/\text{AC}-0.05}$=0.046, and $C/{C}_{0,\text{TiO}2}$=0.081 (Fig. 17b–d).

## Conclusions

In the present study, activated carbon from coffee waste (CWAC-NaOH-800) was used as a substrate to synthesize hybrid adsorbents/photocatalysts (TiO_2_/AC) via the sol–gel method for the synergistic adsorption and photocatalysis of lindane (LIND). To understand in depth the mechanisms involved in the process, TiO_2_/AC composites were synthesized at three TiO_2_/AC ratios by varying the amount of AC (TiO_2_/AC-0.2, TiO_2_/AC-0.1, and TiO_2_/AC-0.05). N_2_ sorption isotherms, MIP data, and SEM images revealed that the meso- and micro-pores of AC resemble parallel capillary tubes penetrating in the AC grains, where the pore sizes are comparable to the throat sizes. With the addition of TiO_2_ nanoparticles in the pore space of AC, pore network effects become evident by creating a micro-porous network with the pore radii ranging from 2.0 to 3.0 nm and the throat radii ranging from 1.2 to 1.4 nm. In the TiO_2_/AC mesoporous network, the pore radii range from 20.0 to 25 nm, while with the mass fraction of TiO_2_ increasing the throat radii decrease from 22.7 to 4–17 nm. The surface area and total pore volume of TiO_2_/AC composites are lower than those of CWAC-NaOH-800, and decrease with the AC mass decreasing, indicating that the micropores of AC were covered or clogged by the TiO_2_ nanoparticles. The XRD pattern of CWAC-NaOH-800 is indicative of its amorphous nature, while TiO_2_ and TiO_2_/AC are highly crystalline with the anatase phase dominating.

The LIND adsorption on CWAC-NaOH-800 is independent on pH, the isotherm is fitted to the monolayer adsorption of Langmuir model with a maximum sorption capacity equal to 9.74 mg/g. Simulating the sorption dynamics with the multi-compartment mass transfer model, it seems that film diffusion and LIND sorption on external surface of AC particles contributes to the total sorption capacity at the early times of the process, and gradually at late times, the molecular diffusion and sorption on the meso-/macro-pores of the adsorbent become dominant, with the surface diffusion and sorption on the micro-pores obtaining a sensible but weaker contribution to the total sorption capacity, at the very late stages of the process. The adsorption mechanism is likely to be the hydrophobic interactions due to the absence of polarization of LIND and CWAC-NaOH-800, as generally ACs due to their hydrophobic properties tend to repel water molecules and preferentially adsorb non-polar or hydrophobic compounds such as LIND.

To investigate the adsorption-photocatalytic capacity of TiO_2_/AC composites, two experimental setups were tested and compared: a UVA oven with LEDs (22 W) and a UVA lamp (6 W) both emitting at 375 nm. The TiO_2_/AC-0.1 composite has comparable adsorption capacity with that of CWAC-NaOH-800, and the simulation of sorption dynamics with the multi-compartment model shows that at early times the contribution fraction of film diffusion and sorption on the outer surface of the particles of TiO_2_/AC-0.1 to the total LIND sorption is higher than the corresponding one of AC, mainly because the presence of agglomerates of TiO_2_ nanoparticles shifts the overall particle size distribution to lower sizes. From kinetic experiments examining the synergistic adsorption-photocatalysis of LIND, it was observed that the TiO_2_/AC composites are more effective than the pure TiO_2_ nanoparticles, which might be attributed to the significant contribution of adsorption onto AC to the overall process along with the enhanced performance of composite phototocatalyst due to the “co-adsorption” effect. The photocatalytic activity of TiO_2_/AC composites was confirmed by measuring the total organic carbon (TOC) during the synergistic adsorption-photocatalysis, while the decrease of pH and increase of redox potential (ORP) during photocatalysis revealed indirectly the oxidation of LIND. Finally, from the inverse modeling of the adsorption-photocatalysis experiments with a model that accounts for the two processes, it was found that the kinetic constant of photocatalysis was maximized for the TiO_2_/AC-0.1 composite in the UVA lamp set-up, due to the better spatial distribution of UV-radiation at the radial direction. The present work could motivate a sustainable two-step strategy for the remediation of polluted water as follows:

(i) Adsorption of pollutants in low-cost porous adsorbents, the development of which will be carried out based on the physicochemical properties of the pollutants.
(ii) In situ regeneration of the saturated adsorbent material through photocatalysis or any other advanced oxidation process. In this manner, the direct oxidation of pollutants with the probable creation of toxic intermediates in water can be avoided and replaced by the repeated use of the regenerated adsorbent material.

## Data Availability

The data that support this study will be shared on request to the corresponding author.
